# The Psychological Effects of Coronavirus Disease 2019 on Health Care Workers in Israel—A Personal Experience

**DOI:** 10.1177/08850666211038890

**Published:** 2021-08-30

**Authors:** Reut Kassif Lerner

**Affiliations:** 1 Department of Pediatric Intensive Care, The Edmond and Lily Safra Children's Hospital, Sheba Medical Center, Ramat Gan, Israel; 2 Tel Aviv University, Tel Aviv-Yafo, Israel

**Keywords:** COVID-19, intensive care, Israel, psychological effects, health-care workers

## Abstract

The pandemic of coronavirus disease 2019 (COVID-19) has had an ongoing global influence with high prevalence of psychological effects on both patients and health-care workers. The effect of anxiety and depression on medical professionals was substantial, as most medical resources have been used for treatment of patients; therefore, the availability of psychological services was insufficient. The situation in Israel was no different, and no society, including my own, was prepared for the psychological toll that COVID-19 has taken on us. This is a narrative of a pediatric intensivist working at the adult COVID-19 critical care unit, and a desperate call to mobilize resources to provide the psychological care that we all need.

The pandemic of coronavirus disease 2019 (COVID-19) has had an ongoing global influence, with high prevalence of psychological effects on both patients and health-care workers. The effect of anxiety and depression on medical professionals was substantial, as most medical resources have been used for treatment of patients; therefore, the availability of psychological services was insufficient.

The situation in Israel was no different. While many might characterize Israel as a nation prepared for missile and biological threats, were we properly prepared to face this horrible pandemic? Perhaps, more so than other locations, Israel's infrastructure is built to be prepared for different attacks. As with every dwelling place, hospitals have underground shelters with prebuilt foundations for hospital wards. Most citizens, including myself, have served in the Israeli Defense Forces (IDF) for several years, and have participated in simulated exercises related to immediate treats such as terror attacks and wars. Therefore, many of the struggles that health-care workers encountered in the early months of the pandemic should not have been issues for health-care workers in Israel. However, no society, including my own, was prepared for the psychological toll that COVID-19 has taken on us.

I am a pediatric intensivist. In March 2020, due to lack of human resources, I was reallocated to work in the new high-tech COVID-19 Adult Intensive Care Unit at Sheba Medical Centre, Israel, which was built 2 floors underground, in a parking lot. No windows, isolated, and far from the known and familiar. This work environment has made me anxious, frustrated, and vulnerable.

I worked as a critical care consultant for patients who were 30 to 50 times the size and weight of the usual pediatric patients. Different drug dosages, different background diseases, different ventilation strategies, and different lab results. I was supported by and collaborated with adult specialty physicians from all aspects—general medicine, cardiology, infectious disease, and cardiac surgery. I had new terms, new language, new perspective and new decision-making processes. I often found myself supporting general physicians in their early training programs, teaching them the basic physiology of respiratory and cardiac interactions, ventilation, and inotropic support. We were all forced to adapt, to grow. Within a few days, the unit reached capacity. I found myself treating patients, including fellow health-care workers, with massive pulmonary emboli, cytokine storms - for which we attempted plasma exchange, or myocarditis - requiring support by extracorporeal membrane oxygenation. The physiology was familiar, but disease presentations and underlying morbidities made me feel somewhat unprepared. The last time I had contact with an adult patient was over a decade ago. However, I have now found that the basics are more or less the same: lung mechanics and cardiac function, extracorporeal support, resuscitation, withdrawal of life support. We encouraged early intubation for all patients who presented with fulminant respiratory failure. We quickly realized many of the patients would require ventilator support for weeks and therefore placed early tracheostomy in those for whom we estimated a good chance of recovering. I found myself in the midst of new and evolving treatment protocols. It felt like medicine of the new era, at times even managed by robots, cameras, and tablets. And yet, in the midst of something that could be intellectually invigorating, it all felt a bit overwelming. Was the intellectual stimulation insufficient to compensate for the mental and emotional strain? We worked 12-hour shifts. We experienced ongoing fatigue and confusion, waking up not knowing whether it is day or night, working in an underground space with no access to sunlight, detached from society and the rest of the hospital. Though we had support from our diverse team of health-care workers, death was still difficult to face on a daily basis. We lost more than half of our patients. I felt depressed—feeling the chances of survival of the next patient were similar to the toss of a coin. The degree of isolation was nearly paralyzing.

To protect ourselves and those we love, we were forced to practice medicine at a distance, touching our patients via 3 layers of gloves, full-face shields and protective coverings over our heads and bodies. We conducted video conferences with families with no visitations allowed. The loneliness was very real. As a pediatrician, mother, and granddaughter, I tried to balance professionalism, empathy, and the anxiety of dealing with a highly infectious contagion—constantly wondering if I was exposing my family. By 3 months that I worked in the COVID unit, despite my country's preparedness and my experience in the IDF, I mainly struggled with isolation and uncertainty.

I hope that the current vaccines and treatments, as well as those under development, will be effective at curbing the most severe physical manifestations of the disease. Now we must face what is underneath—thousands, if not hundreds of thousands of health-care workers, no matter what their background going into the pandemic, are struggling with the psychological effects of their practice of medicine.

Exactly one year later, going back to the shelter, now as a consultant pediatric intensivist looking after criticality ill pediatric patients, my comfort zone. An outbreak of violence in the ongoing Israeli–Palestinian conflict commenced on May 10, 2021, exposed the general population to rocket attacks necessitating us to go underground. And again, feelings of anxiety yet familiarity have emerged, depression and seclusion, facing situations that were far suppressed and forgotten. Probably not for the last time. Doctors, paramedical personnel, experiencing symptoms of posttraumatic stress disorder: vivid flashbacks and intrusive thoughts, paralyzing at times.

Would things have been different having proper psychological support? We must be prepared for what lies ahead. Let this be a call to mobilize resources to provide the care that we all need, not just health-care workers, but also nonmedicals which probably face similar psychological effects.

